# Elevated Systemic Inflammation Response Index on Admission Indicates Stress Ulcer Risk in Acute Ischemic Stroke

**DOI:** 10.1002/brb3.71867

**Published:** 2026-11-11

**Authors:** Yang Yang, Liping Yan, Guojuan Chen, Peng Ding, Ling Ling, Fanshu Zhao, Wei Yue

**Affiliations:** ^1^ Department of Neurology Clinical College of Neurology, Neurosurgery, and Neurorehabilitation, Tianjin Medical University Tianjin China; ^2^ Department of Neurology Tianjin Huanhu Hospital Tianjin China; ^3^ Tianjin Key Laboratory of Cerebral Vascular and Neurodegenerative Diseases Tianjin Huanhu Hospital Tianjin China; ^4^ Department of Neurology Tangshan Gongren Hospital Tangshan China; ^5^ Department of Neurology Tianjin Union Medical Center Tianjin China; ^6^ Department of Neurology Tangshan Hongci Hospital Tangshan China

**Keywords:** acute ischemic stroke, association, stress ulcer, systemic inflammation, systemic inflammation response index

## Abstract

**Background:**

Stress ulcers following acute ischemic stroke worsen prognosis and increase healthcare burden. Inflammatory mechanisms are involved, yet the association between the systemic inflammation response index (SIRI) and ulcer risk remains unclear. This study investigates the correlation between SIRI levels and post‐stroke stress ulcer risk.

**Methods:**

We analyzed data from a prospective registry of adult stroke patients admitted within seven days of onset. SIRI was defined as neutrophil count × monocyte count / lymphocyte count. SIRI levels were categorized into quartiles (Q1–Q4). Using the lowest quartile as reference, Cox regression models with full adjustment for confounders assessed the hazard ratios for ulcer risk across SIRI groups. Restricted cubic spline and subgroup analyses were employed.

**Results:**

Among 1,171 patients, 86 (7.3%) developed stress ulcers within 7 days. After full adjustment, higher SIRI levels were independently associated with increased risk, with adjusted hazard ratios of 3.17 (95% CI: 1.06–9.50) for Q3 and 5.61 (95% CI: 1.93–16.33) for Q4. A linear dose–response relationship was observed, with risk rising notably when SIRI exceeded 1.1. This association was modified by age, NIHSS score, and infarct location.

**Conclusions:**

Elevated SIRI levels are significantly associated with an increased risk of post‐stroke stress ulcers. As an easily accessible biomarker, it can be used to identify high‐risk patients at an early stage, thereby guiding targeted prevention strategies.

## Graphical abstract

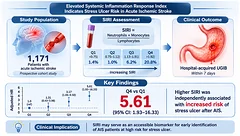

**Abbreviations**
APTT: activated partial thromboplastin time
CI: confidence interval
GCS: Glasgow Coma Scale
HR: hazard ratio
IQR: inter‐quartile range
NIHSS: National Institutes of Health Stroke Scale
SD: standard deviation
TOAST: Trial of ORG 10172 in acute stroke treatment

## 1 Introduction

Research confirms that patients with a range of neurological conditions, such as ischemic stroke, traumatic brain injury, spinal cord injury (SCI), intracerebral hemorrhage, and central nervous system tumors, exhibit a significantly higher incidence of stress ulcers compared to patients without neurological disorders. Risk factors such as age >60 years, mechanical ventilation, elevated plasma cortisol levels, and cerebral vasospasm have been demonstrated to increase the risk of stress ulcers following stroke (Venkatesan and Lucke‐Wold 2025). Cushing ulcer, also known as neurogenic ulcer, may be defined as gastroduodenal ulceration associated with acutely raised intracranial pressure, typically in the context of cerebrovascular accidents such as brain tumors, ischemic stroke, and intracranial surgery (Kemp et al. 2015). The pathophysiological mechanism underlying stress ulcers following ischemic stroke is multifactorial, characterized by an altered balance between protective and destructive factors regulating the gastric mucosa. Destructive factors include excessive secretion of gastric acid and pepsin, which themselves result from uncontrolled vagal activity and diminished sympathetic drive (Gudeman et al. 1983, Chan et al. 1989, Metz et al. 1993, Li et al. 2010, Ali et al. 2021). The pathophysiology of stress ulcers is also attributed to reduced blood flow to the abdomen, which in turn leads to diminished mucosal defense against gastric acid (Moran et al. 1987). Furthermore, the pathophysiological mechanisms underlying stress‐related injuries remain unclear. For example, Batts and colleagues retrospectively collected inpatient records and compiled endoscopy findings to investigate risk factors for these complications (Wijdicks et al. 1994). Substances such as acetylcholine, histamine, and endogenous thyrotropin‐releasing hormone may also increase the vulnerability of the mucosa (Bresalier 1991).

The SIRI, based on peripheral blood cell counts, combines neutrophil (NEUT), monocyte (MONO), and lymphocyte (LY) levels to assess systemic inflammation as an inflammatory biomarker. SIRI = Neutrophil count × Monocyte count / Lymphocyte count; therefore, SIRI reflects both the significant pro‐inflammatory response caused by MONO and the anti‐inflammatory response mediated by LY (Yi et al. 2021). As a novel biomarker, SIRI is widely used to predict disease prognosis (Ji et al. 2022)_._ Inflammation is closely linked to the pathogenesis of stroke. Ischemia triggers local immune responses and inflammatory cytokine production, thereby disrupting the tight junctions of the blood‐brain barrier (Kim et al. 2016). The neutrophil‐lymphocyte ratio (NLR), platelet‐lymphocyte ratio (PLR), prognostic nutritional index, and lymphocyte‐to‐monocyte ratio have been extensively employed to predict adverse outcomes and complications in ischemic stroke (Petrovic‐Djergovic et al. 2016). Chen et al. reported that the combined use of NLR and PLR is more effective than either alone in predicting poor outcomes after ischemic stroke (Pikija et al. 2018, Ferro et al. 2021, Lee et al. 2021, Xiang et al. 2020, Gong et al. 2021, Chen et al. 2020). Current research on post‐stroke stress ulcers primarily focuses on developing predictive models for high‐risk factors and validating the efficacy of pharmacological interventions. However, studies investigating how SIRI contributes to the inflammatory response leading to post‐stroke stress ulcers remain limited. It remains uncertain whether alterations in SIRI levels influence the risk of developing post‐stroke stress ulcers.

Therefore, this study aims to utilize data from the SPARK cohort (Study of the Impact of Cardiac Function on Short‐Term Functional Outcomes in Patients with Acute Ischemic Stroke) to investigate and evaluate whether SIRI levels and changes are associated with the incidence of stress‐induced ulcers following stroke.

## 2 Materials and Methods

### 2.1 Study Design

Patients diagnosed with AIS were recruited at the Stroke Center of Tianjin Huanhu Hospital between January 19 and March 20, 2023. Inclusion criteria were as follows: (1) Age ≥ 18 years; (2) Time from symptom onset to hospitalization ≤ 7 days; (3) clinically diagnosed ischemic stroke causing assessable neurological deficits (e.g., language, motor function, cognitive impairment, gaze abnormality, visual field defect, or visual neglect), with head MRI including diffusion‐weighted imaging (DWI) performed within 48 h of symptom onset, confirming the presence of acute ischemic infarcts consistent with the clinical symptoms, characterized by high signal intensity on DWI sequences and low signal intensity on ADC sequences; (4) Echocardiography completed during hospitalization, with time from admission to echocardiography ≤ 7 days. Exclusion criteria were as follows: (1) Transient ischemic attack; (2) Diagnosis of hemorrhage or other pathological conditions based on head CT and/or MRI, such as vascular malformations, tumors, abscesses, multiple sclerosis, or other common non‐ischemic diseases; (3) Expected survival < 6 months (e.g., due to malignant tumors, severe cardiopulmonary disease); (4) Participation in other interventional clinical studies that may influence outcome assessment, with informed consent already obtained. All study participants provided informed consent, and the research received ethical approval from the Medical Research Ethics Committee of Tianjin Huanhu Hospital and the Tianjin Municipal Health Bureau.

### 2.2 Data Collection

We first screened the enrolled population to identify patients with acute ischemic stroke complicated by stress ulcers. We collected and recorded their baseline information, including demographic details (name, gender, age, etc.), past medical history (hypertension, coronary heart disease, diabetes), alcohol consumption history, laboratory tests (platelet count, APTT, serum creatinine, fasting blood glucose, serum total bilirubin, and creatine kinase), and hospitalization course (oral aspirin therapy, mechanical ventilation, endovascular treatment, and anticoagulation therapy). Subsequently, a neurologist conducted a comprehensive physical examination to assess and record their heart rate, systolic blood pressure, National Institutes of Health Stroke Scale on admission, Glasgow Coma Scale on admission, TOAST stroke subtypes (large‐artery atherosclerosis, cardioembolism, small‐artery occlusion, other etiology, or undetermined etiology), and determined the location of cerebral infarction via CT/MRI (massive cerebral infarction, insular infarction, cerebellar infarction, medullary infarction, pontine infarction, and midbrain infarction) and infarct distribution (anterior circulation and posterior circulation). Subsequently, SIRI was calculated from blood count data (NEUT, MONO, and LY). We then examined the correlation between SIRI levels and the incidence of stress‐induced ulcers post‐stroke across these subgroups.

### 2.3 Diagnosis of Stress Ulcer

In the SPARK study, stress ulcers were strictly defined as hospital‐acquired upper gastrointestinal bleeding events occurring after stroke onset within 7 days. Patients were prospectively monitored on a daily basis during hospitalization for clinical signs of gastrointestinal bleeding. The diagnostic criteria were based on a 2024 guideline developed jointly by the Society of Critical Care Medicine (SCCM) and the American Society of Health‐System Pharmacists (ASHP). The guideline systematically standardizes the definition, clinical criteria, and research framework for stress‐related mucosal disorders (SRMD) (Maclaren et al. 2024). The guideline explicitly states that the definition of stress ulcers is based on the following points: (1) overt upper gastrointestinal bleeding manifestations, including hematemesis, bloody nasogastric aspirate, melena, or hemodynamic instability requiring transfusion or surgical intervention after exclusion of other causes; (2) endoscopically confirmed lesions associated with upper gastrointestinal bleeding, including gastric, or duodenal erosions, superficial ulcers, and diffuse mucosal hemorrhage, as supporting evidence. The onset time of stress ulcer was defined as the interval between stroke onset and the first documented evidence of gastrointestinal bleeding, ensuring consistency and reducing potential detection bias in outcome assessment.

### 2.4 Statistical Analysis

In this study, we divided SIRI levels into four quartiles using the quartile method: Q1 (SIRI < 0.75), Q2 (SIRI = 0.75–1.12), Q3 (SIRI = 1.13–1.91), and Q4 (SIRI > 1.92). At baseline, this allowed us to describe in detail how characteristics of different populations influenced the association between SIRI levels and post‐stroke stress ulcers. Cox regression analysis was employed to establish three regression models: Model 1 adjusted for gender and age; Model 2 adjusted for gender, age, and the National Institutes of Health Stroke Scale; Model 3 adjusted for age, gender, the National Institutes of Health Stroke Scale, the Glasgow Coma Scale, insular cortex infarction, medullary infarction, cerebellar infarction, massive cerebral infarction, endovascular treatment, and mechanical ventilation. Through Cox regression analysis, we controlled the interference of other confounding factors, reduced confounding bias, and more clearly determined the impact of SIRI levels on stress‐induced ulcers. Subsequently, we conducted stratified subgroup analyses based on gender, age, National Institutes of Health Stroke Scale, and infarct location to assess whether SIRI's impact on stress ulcer risk varied across different demographics, including gender, age, stroke severity, and infarct location. The RCS curve was used to observe whether there was a nonlinear relationship between SIRI levels and the risk of developing stress ulcers. All tests were two‐sided, and p < 0.05 was considered statistically significant.

## 3 Results

### 3.1 Baseline Characteristics of Study Participants

The study flowchart is presented in Figure 1. Initially, 1357 AIS patients were screened, of which 186 were excluded due to missing blood count data, yielding a final analytic cohort of 1171 patients. A total of 86 patients (7.3%) developed stress ulcers within 7 days after AIS. Among patients who developed stress ulcers, 69 cases (5.9%) occurred within 3 days of stroke onset, while 17 cases (1.4%) occurred between 3 and 7 days, indicating a predominantly early‐onset pattern.

**FIGURE 1:**
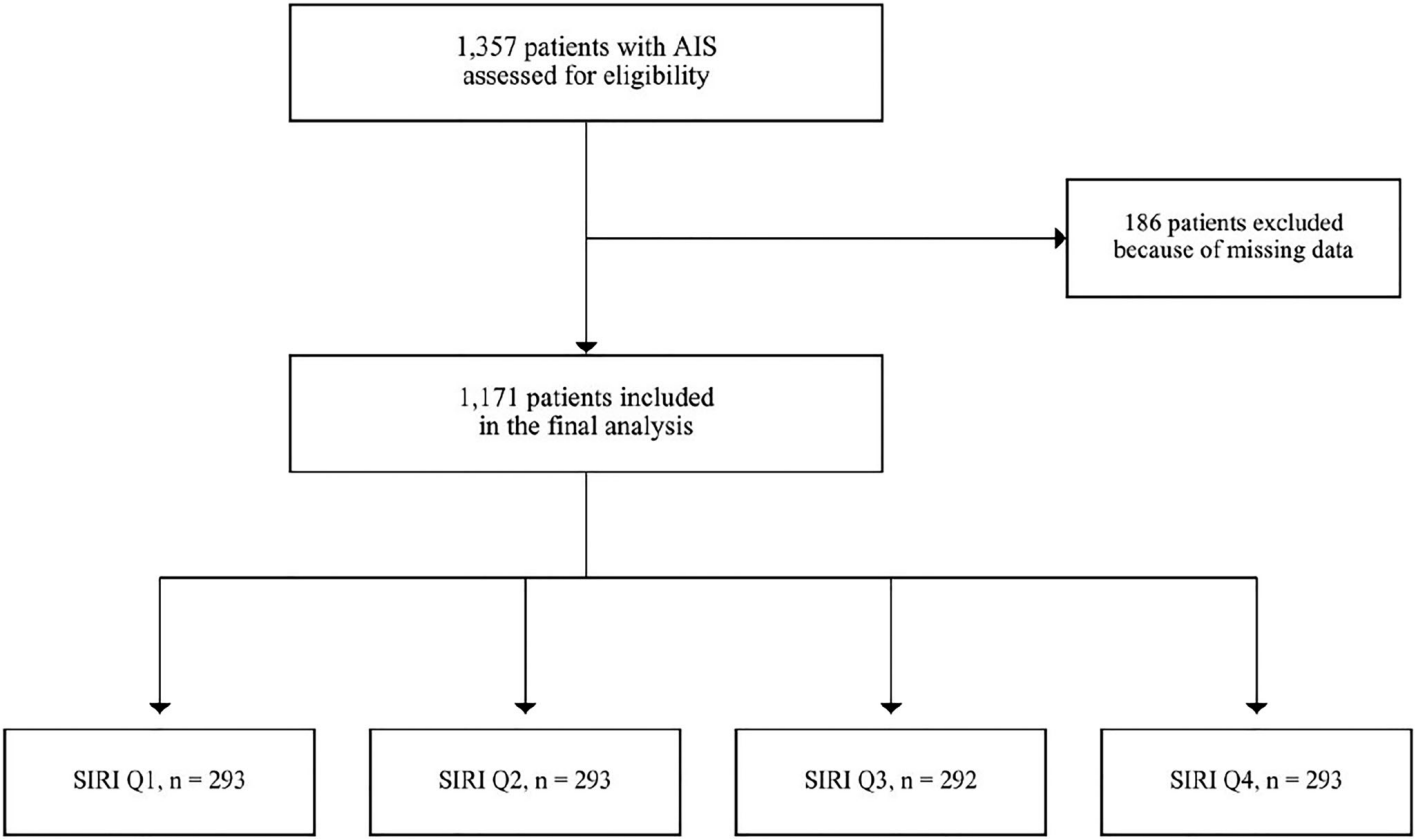
Flowchart of the study population. A total of 1,357 patients with acute ischemic stroke (AIS) who were admitted within 7 days of stroke onset were initially screened. After excluding 186 patients with missing data, 1,171 patients were included in the final analysis. Based on the quartiles of the systemic inflammation response index (SIRI), the included patients were divided into four groups: Q1 (lowest, n = 293), Q2 (n = 293), Q3 (n = 292), and Q4 (highest, n = 293). SIRI quartile cutoffs were determined according to the distribution of SIRI in the study population (Q1 ≤ 25th percentile, Q2 25th–50th, Q3 50th–75th, and Q4 ≥ 75th percentile). Abbreviations: AIS, acute ischemic stroke; SIRI, systemic inflammation response index; Q1, SIRI < 0.75; Q2, SIRI 0.75–1.12; Q3, SIRI 1.13–1.91; Q4, SIRI > 1.92; n, number of patients.

**FIGURE 2:**
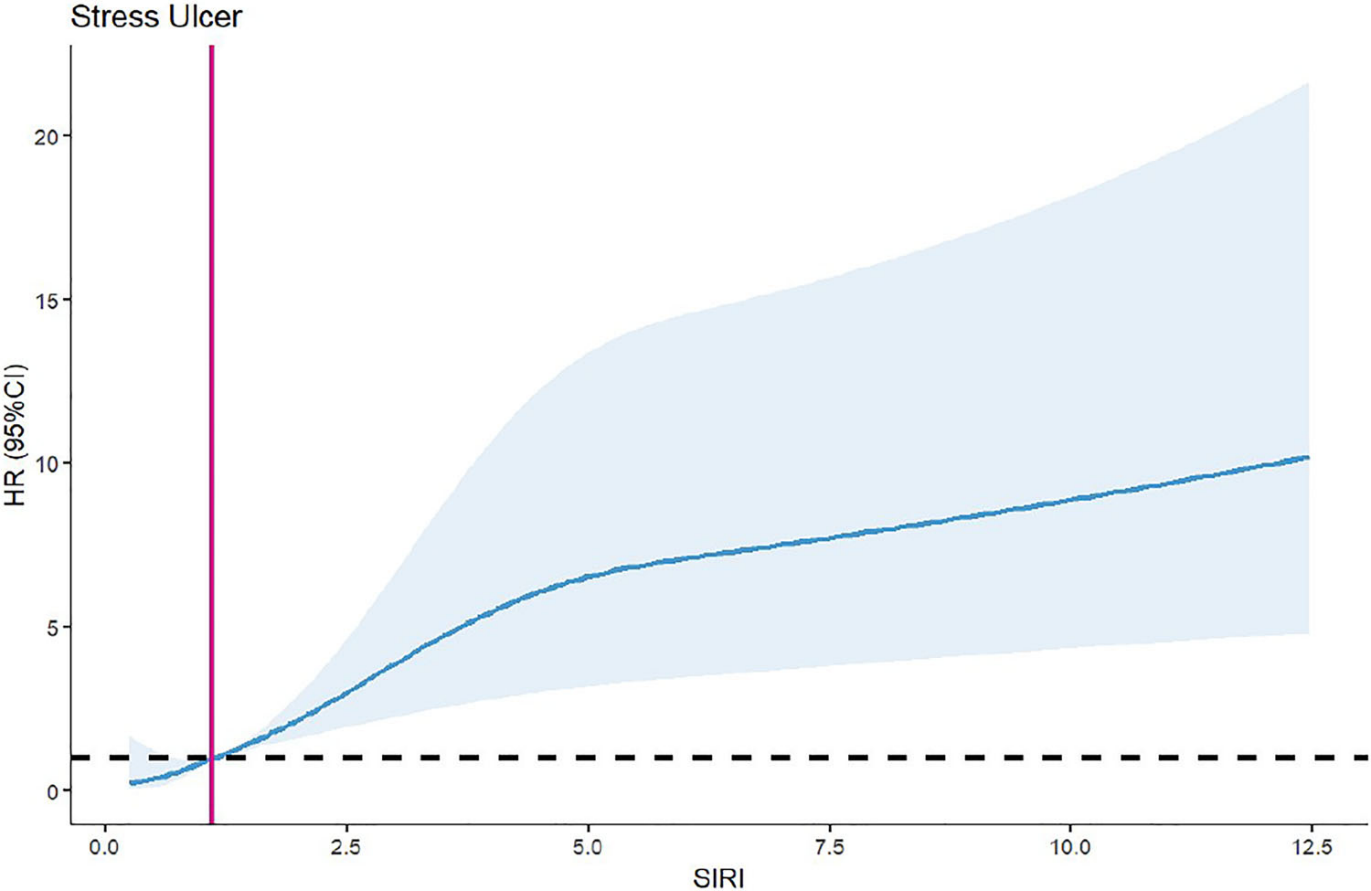
Restricted cubic spline (RCS) analysis of the association between SIRI and the risk of stress ulcer. RCS analysis of the association between the SIRI and stress ulcer risk. The solid blue line represents the estimated hazard ratio (HR), and the shaded area represents the 95% CI. The horizontal dashed line indicates HR = 1, and the vertical line indicates the reference value of SIRI. Abbreviations: RCS, restricted cubic spline; SIRI, systemic inflammation response index; HR, hazard ratio; CI, confidence interval.

The baseline characteristics of the included patients are summarized in Table 1. The mean age was 64 (±12) years, and 74.7% were male. Compared with Q4, the other quartiles showed a lower prevalence of hypertension and coronary heart disease, but a higher prevalence of diabetes. Fewer patients received mechanical ventilation, anticoagulation therapy, or endovascular treatment. Lower platelet counts and lower levels of serum creatinine, serum total bilirubin, and creatine kinase were observed. Lower incidences of massive cerebral infarction and insular, medullary, and midbrain infarction were noted. The proportions of the large‐artery atherosclerosis and cardioembolism TOAST subtypes were also lower.

**TABLE 1:** Baseline characteristics of the patients by quartiles. (n=1171).

Variables	Total (n = 1171)	Q1 (<0.75) n = 293	Q2 (0.75‐1.12) n = 293	Q3 (1.13‐1.91) n = 292	Q4 (>1.92) n = 293	*p* value
Stress ulcer, n	86 (7.3%)	4 (1.4%)	3 (1.0%)	18 (6.2%)	61 (20.8%)	<0.001
Sex, male, n	867 (74.0%)	172 (58.7%)	226 (77.1%)	238 (81.5%)	231 (78.8%)	<0.001
Age, mean (SD), years	64 (±12)	63 (±11)	63 (±11)	63 (±12)	66 (±12)	<0.001
Hypertension, n	903 (77.1%)	207 (70.6%)	232 (79.2%)	223 (76.2%)	241 (82.3%)	0.007
Diabetes, n	441 (37.7%)	114 (38.9%)	105 (35.8%)	118 (40.4%)	104 (35.5%)	0.545
Coronary heart disease, n	196 (16.7%)	40 (13.7%)	36 (12.3%)	49 (16.8%)	71 (24.2%)	<0.001
Drinking, n	362 (30.9%)	78 (26.6%)	88 (30.0%)	109 (37.3%)	87 (29.7%)	0.037
NIHSS score on admission, median (IQR)	5 (2−9)	4 (2−7)	4 (2−7)	5 (2−8)	9 (4−15)	<0.001
GCS score on admission, median (IQR)	15 (15−15)	15 (15−15)	15 (15−15)	15 (15−15)	15 (14−15)	<0.001
Systolic blood pressure, mean (SD), mmHg	151 (±24)	147 (±24)	148 (±22)	154 (±24)	156 (±25)	<0.001
Heart rate on admission, mean (SD), bmp	73 (±14)	69 (±14)	71 (±11)	74 (±14)	79 (±17)	<0.001
Mechanical ventilation, n	16 (1.4%)	1 (0.3%)	0 (0.0%)	0 (0.0%)	15 (5.1%)	<0.001
Anticoagulant therapy, n	269 (23.0%)	58 (19.8%)	55 (18.8%)	73 (25.0%)	83 (28.3%)	0.018
Oral aspirin therapy, n	1110 (94.8%)	283 (96.6%)	277 (94.5%)	280 (95.9%)	270 (92.2%)	0.078
Endovascular treatment, n	16 (1.4%)	1 (0.3%)	0 (0.0%)	1 (0.3%)	14 (4.8%)	<0.001
Intravenous thrombolysis, n	77 (6.6%)	13 (4.4%)	14 (4.8%)	26 (8.9%)	24 (8.2%)	0.056
Platelet count, mean (SD), ×10^9^/L	228 (±64)	217 (±55)	226 (±59)	230 (±60)	240 (±77)	0.020
APTT, mean (SD), sec	24.8 (±4.2)	24 (±4.2)	24 (±3.9)	25 (±3.7)	25 (±4.7)	<0.001
Fasting blood glucose, median (IQR), mmol/L	5.77 (4.94−7.80)	5.67 (4.97−8.01)	5.54 (4.75−7.22)	5.95 (4.93−8.02)	6.02 (5.07−8.22)	0.058
Serum creatinine, median (IQR), µmol/L	71.40 (±26.28)	66.70 (±27.50)	72.00 (±20.38)	72.05 (±24.50)	74.60 (±30.80)	<0.001
Serum total bilirubin, median (IQR), µmol/L	13.71 (±7.39)	13.63 (±6.43)	13.38 (±6.13)	13.80 (±7.08)	14.15 (±9.37)	0.495
Creatine kinase, median (IQR), U/L	81 (54−‐143)	70 (52−110)	72 (50−126)	84 (58−135)	101 (62−224)	<0.001
Massive cerebral infarction, n	93 (7.9%)	1 (0.3%)	7 (2.4%)	15 (5.1%)	70 (23.9%)	<0.001
Insular cortex infarction, n	135 (11.5%)	12 (4.1%)	17 (5.8%)	25 (8.6%)	81 (27.6%)	<0.001
Infarction location, n						
Anterior circulation	843 (72.0%)	225 (75.8%)	214 (73.0%)	197 (67.5%)	207 (70.6%)	0.081
Posterior circulation	328 (28.0%)	68 (23.2%)	79 (27.0%)	95 (32.5%)	86 (29.4%)	0.081
Medullary infarction, n	45 (3.8%)	7 (2.4%)	9 (3.1%)	11 (3.8%)	18 (6.1%)	<0.001
Pontine infarction, n	77 (6.6%)	17 (5.8%)	24 (8.2%)	16 (5.5%)	20 (6.8%)	0.546
Midbrain infarction, n	21 (1.8%)	5 (1.7%)	2 (0.7%)	6 (2.1%)	8 (2.7%)	0.303
Cerebellar infarction, n	147 (12.6%)	15 (5.1%)	30 (10.2%)	53 (18.2%)	49 (16.7%)	<0.001
TOAST stroke subtypes, n						<0.001
Large‐artery atherosclerosis	596 (50.9%)	123 (42%)	137 (46.8%)	160 (54.8%)	176 (60.1%)	
Cardioembolism	91 (7.8%)	11 (3.8%)	15 (5.1%)	17 (5.8%)	48 (16.4%)	
Small‐artery occlusion	257 (21.9%)	85 (29.0%)	76 (25.9%)	72 (24.7%)	24 (8.2%)	
Other etiology	21 (1.8%)	7 (2.4%)	5 (1.7%)	2 (0.7%)	7 (2.4%)	
Undetermined	206 (17.6%)	67 (22.9%)	60 (20.5%)	41 (14.0%)	38 (13.0%)	

**Abbreviations**: APTT = Activated Partial Thromboplastin Time; GCS = Glasgow Coma Scale; IQR = Inter‐Quartile Range; NIHSS = National Institutes of Health Stroke Scale; SD = Standard Deviation; TOAST = Trial of ORG 10172 in Acute Stroke Treatment.

### 3.2 SIRI Level and Stress Ulcer Risk

To assess the independent association between the SIRI score and the risk of developing stress ulcers, we conducted a Cox regression analysis. The Cox regression analysis presented in Table 2 shows that the risk of stress ulcers increases progressively with higher SIRI quartiles. In the crude analysis (unadjusted for confounders), compared with Q1, the hazard ratio (HR) for stress ulcer occurrence in Q2 was 0.75 (95% CI: 0.17–3.34, *p* = 0.704), Q3 showed HR = 4.64 (95% CI: 1.57–13.70, *p* = 0.006), and Q4 exhibited HR = 16.48 (95% CI: 5.99–45.33, *p* < 0.001), with Q4 demonstrating significantly higher stress ulcer risk than Q2 and Q3. Q4 demonstrated the poorest SIRI control and the highest risk of stress ulcer (HR = 16.48, 95% CI: 5.99–45.33). In the fully adjusted model (Model 3), the hazard ratio (HR) for Q4 was 5.61 (95% CI: 1.93–16.33, *p* = 0.002), indicating that patients with the highest SIRI levels had more than a fivefold higher risk of developing stress ulcers compared with those in Q1. Similarly, patients in Q3 also showed a significantly increased risk (HR = 3.17, 95% CI: 1.06–9.50, *p* = 0.004), whereas no significant association was observed in Q2. Regardless of confounding factor adjustment, Q2 consistently showed no significant association with stress ulcer incidence (*p* > 0.05). These findings suggest a dose–response relationship between SIRI levels and stress ulcer risk, with higher SIRI levels associated with progressively increased risk.

**TABLE 2:** Cox regression analysis for the association between SIRI quartiles and stress ulcer.

	Crude		Model 1		Model 2		Model 3	
		*p*‐value	HR (95%CI)	*p*‐value	HR (95%CI)	*p*‐value	HR (95%CI)	*p*‐value
Q 1	Ref.		Ref.		Ref.		Ref.	
Q 2	0.75 (0.17−3.34)	0.704	0.75 (0.17−3.37)	0.710	0.71 (0.16−3.20)	0.659	0.66 (0.15−2.96)	0.589
Q 3	4.64 (1.57−13.70)	0.006	4.69 (1.58−13.96)	0.005	3.90 (1.31−11.62)	0.015	3.17 (1.06−9.50)	0.004
Q 4	16.48 (5.99−45.33)	<0.001	16.16 (5.82−44.84)	<0.001	8.74 (3.06−24.98)	<0.001	5.61 (1.93−16.33)	0.002

***Note***: Model 1 adjusted by age, gender; Model 2 adjusted by age, gender, national institutes of health stroke scale; Model 3 adjusted by age, gender, national institutes of health stroke scale, glasgow coma scale, insular cortex infarction, medullary infarction, cerebellar infarction, massive cerebral infarction, endovascular treatment, mechanical ventilation.

**Abbreviations**: a CI not including 1 indicates statistical significance; CI, confidence interval; HR represents the relative risk of stress ulcer occurrence in each group compared with the reference group (Q1). An HR > 1 indicates increased risk, whereas HR < 1 indicates decreased risk. The 95% CI reflects the precision of the estimate; HR, hazard ratio; NIHSS, National Institutes of Health Stroke Scale; SIRI, systemic inflammatory response index.

Restricted cubic spline (RCS) analysis was performed to further assess the dose–response relationship between SIRI and stress ulcer risk. SIRI showed an approximately linear association with stress ulcer risk (*P* for nonlinearity > 0.05). As illustrated in Figure 2, the results showed that, as SIRI levels increased, the overall risk of stress ulcers exhibited a continuous upward trend. The risk increased markedly at SIRI values between 1.1 and 5.0 before gradually leveling off beyond 5.0. No distinct U‐ or J‐shaped relationship was observed in the curve, suggesting that the relationship between SIRI and the risk of stress ulcers is primarily positive. Elevated SIRI levels significantly increased the risk of stress ulcers in acute ischemic stroke.

### 3.3 Subgroup Analyses

The subgroup analyses stratified by sex, age, NIHSS severity on admission, and infarct location are presented in Figure 3. Overall, higher SIRI quartiles were generally associated with an increased risk of stress ulcers across the prespecified subgroups, although the magnitude and precision of the associations varied. Statistically significant interactions were observed for sex, age, NIHSS severity, and infarct location.

**FIGURE 3:**
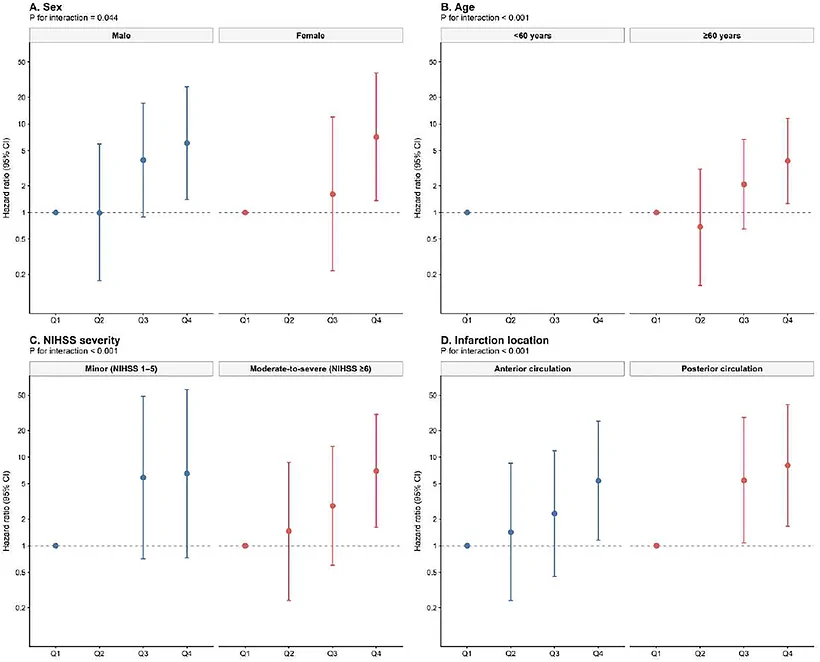
Subgroup analyses of the association between SIRI quartiles and stress ulcer risk in patients with acute ischemic stroke. HRs and 95% CI for stress ulcer risk across SIRI quartiles are presented according to sex (A), age (B), NIHSS severity on admission (C), and infarct location (D). Circles represent adjusted HRs, and vertical error bars represent 95% CI. Q1 was used as the reference group (HR = 1), and the horizontal dashed line indicates HR = 1. Estimates are not shown when they could not be reliably calculated. P values are for interaction. Abbreviations: SIRI, systemic inflammatory response index; NIHSS, National Institutes of Health Stroke Scale; HR, hazard ratio; CI, confidence interval.

In the sex‐stratified analysis, patients in Q4 had a higher risk of stress ulcers than those in Q1 among both males (HR: 6.08, 95% CI: 1.40–26.27) and females (HR: 7.13, 95% CI: 1.36–37.52), with evidence of an interaction between sex and SIRI quartiles (*P* for interaction = 0.044). In the age‐stratified analysis, the Q4 versus Q1 association was statistically significant among patients aged ≥60 years (HR: 3.82, 95% CI: 1.26–11.61), whereas the corresponding estimate among patients aged <60 years could not be reliably calculated (*P* for interaction <0.001). Similarly, Q4 was associated with an increased risk of stress ulcers among patients with moderate‐to‐severe stroke (NIHSS ≥6; HR: 6.98, 95% CI: 1.61–30.23), whereas the association did not reach statistical significance among those with minor stroke (NIHSS 1–5; HR: 6.52, 95% CI: 0.73–57.92; P for interaction <0.001). When stratified by infarct location, the Q4 versus Q1 association remained statistically significant in both the anterior circulation (HR: 5.42, 95% CI: 1.16–25.42) and posterior circulation (HR: 8.07, 95% CI: 1.66–39.15) subgroups (*P* for interaction <0.001).

## 4 Discussion

In this prospective cohort study, elevated SIRI levels were significantly associated with an increased risk of early stress ulcer development in patients with AIS. Patients in the highest SIRI quartile (Q4) had a more than fivefold higher risk of stress ulcer than those in the lowest quartile, and this association remained statistically significant after multivariable adjustment, including adjustment for stroke severity and other relevant confounders. RCS analysis further supported an association between increasing SIRI levels and stress ulcer risk, while subgroup analyses suggested potential heterogeneity across selected clinical characteristics. Taken together, these findings suggest that SIRI may provide complementary information for identifying patients at increased risk of stress ulcer after AIS, although it should be interpreted as an integrated marker of systemic immune‐inflammatory activation and overall disease burden rather than as a stress‐ulcer‐specific or causally independent predictor.

The association between SIRI and stress ulcer risk may be understood in the context of the systemic immune‐inflammatory response following AIS. Notably, most stress ulcers in our cohort occurred within the first 3 days after stroke onset, which is consistent with the potential involvement of early systemic inflammatory responses in their development. Previous studies have shown that an elevated neutrophil‐to‐lymphocyte ratio (NLR) reflects an increased inflammatory burden, characterized by relative neutrophilia and lymphopenia (Świtońska et al. 2019). Acute cerebral ischemia and tissue injury trigger a complex neuroimmune response involving both the ischemic brain and the peripheral immune system. Damage‐associated signals released from injured tissue activate resident microglia and promote the recruitment and activation of peripheral immune cells, including neutrophils and monocytes/macrophages (Xu et al. 2025, Gong et al. 2024). Concurrently, stroke‐induced immunosuppression is characterized in part by alterations in circulating lymphocyte populations and impaired adaptive immune responses (Guo et al. 2026). Thus, the post‐stroke immune response involves the coexistence of innate inflammatory activation and adaptive immune dysregulation. Compared with NLR, SIRI additionally incorporates monocyte counts and may therefore provide a broader representation of the systemic immune‐inflammatory response following AIS.

Beyond systemic immune dysregulation, disruption of the brain–gut axis may provide an additional link between AIS and gastrointestinal injury. Ischemic stroke can perturb bidirectional brain–gut communication through autonomic and neuroimmune pathways. Post‐stroke alterations in sympathetic and parasympathetic signaling may affect gastrointestinal motility, mucosal perfusion, epithelial homeostasis, intestinal permeability, and local immune responses, thereby increasing gastrointestinal vulnerability during the acute phase of stroke (Wang et al. 2025, Xie et al. 2024, Wang et al. 2024). Recent experimental and translational studies have further demonstrated post‐stroke disruption of intestinal barrier integrity, accompanied by alterations in epithelial tight‐junction proteins, gut microbial composition, and systemic inflammatory signaling (Han et al. 2023, Deng et al. 2024). These immune and autonomic alterations are increasingly recognized as interconnected components of post‐stroke brain–gut axis dysfunction rather than isolated processes, and their interaction may contribute to intestinal barrier impairment, dysbiosis, and gastrointestinal vulnerability following ischemic stroke (Xie et al. 2024, Wang et al. 2024, Deng et al. 2024, Agarwal et al. 2024). In this context, the association between elevated SIRI and stress ulcer risk observed in our study may, at least in part, reflect the broader systemic immune‐inflammatory and brain–gut disturbances accompanying AIS. However, SIRI should be interpreted as an integrated marker of these processes and overall disease burden rather than as evidence of a specific causal pathway leading to stress ulcer formation.

Our findings are consistent with the growing body of evidence regarding the clinical relevance of SIRI in patients with stroke. Zhang et al. reported that elevated SIRI levels were associated with adverse clinical outcomes following intravenous thrombolysis in patients with mild AIS (Chu et al. 2023), while Huang et al. demonstrated an association between higher SIRI levels and greater stroke severity (Huang 2022). Previous studies have also consistently reported associations between elevated SIRI levels and unfavorable functional outcomes in patients with AIS, suggesting that SIRI may provide complementary information for clinical risk stratification (Zhou et al. 2022, Chen et al. 2023, Han et al. 2023). However, the association between SIRI and post‐stroke stress ulcers remains insufficiently characterized. Previous studies of post‐stroke stress ulcers have focused primarily on conventional clinical factors and infarct characteristics; for example, insular infarction has been associated with stress ulcer occurrence during hospitalization (Ding et al. 2024). Our study extends this evidence by demonstrating an association between admission SIRI levels and early stress ulcer risk after AIS, suggesting that SIRI may provide complementary information for identifying patients at increased risk.

Beyond the overall association, subgroup analyses were performed to explore potential heterogeneity in the association between SIRI and stress ulcer risk across sex, age, stroke severity, and infarct location. However, given the limited number of events in some subgroups and the multiple comparisons performed, these findings should be interpreted cautiously and regarded as exploratory and hypothesis‐generating rather than confirmatory.

From a broader clinical perspective, SIRI is readily derived from routine blood cell counts and may provide complementary information for early risk stratification when considered alongside established clinical indicators such as stroke severity and infarct characteristics. Patients identified as being at higher risk may warrant closer gastrointestinal monitoring and careful assessment of established indications for stress ulcer prophylaxis (Alhazzani et al. 2018). However, SIRI should not be used in isolation to guide prophylactic treatment decisions, and its incremental clinical value beyond established risk factors requires prospective external validation.

Our study also has limitations: (1) Our data sources are relatively limited, with a small sample size and insufficient geographical representation, meaning results may only reflect conditions in one region or center. (2) The study population is entirely from China, making the findings non‐generalizable to other countries. Future multicenter studies with larger sample sizes are needed to further validate the accuracy and reliability of these results. (3) The single‐center design and relatively short enrollment period of this study may limit the generalizability of the findings; therefore, external validation in a multicenter cohort is warranted. Furthermore, as data on prophylactic proton pump inhibitor use and detailed nutritional status were not systematically collected, residual confounding cannot be excluded. (4) In addition, the specific diagnostic modality used for each stress ulcer event was not systematically recorded, and the proportion of cases confirmed by endoscopy could not be determined, which may have introduced heterogeneity in outcome ascertainment. (5) SIRI is closely associated with stroke severity markers, including NIHSS score and infarct size; therefore, residual confounding cannot be excluded despite multivariable adjustment. Accordingly, SIRI should be interpreted as an integrated marker of systemic immune‐inflammatory activation and overall disease burden rather than as a stress‐ulcer‐specific predictor. (6) The subgroup analyses were limited by small sample sizes and few stress ulcer events in some strata, resulting in wide confidence intervals and reduced statistical precision. Moreover, multiple comparisons without adjustment for multiplicity may have increased the risk of type I error. Therefore, these findings should be considered exploratory and hypothesis‐generating rather than confirmatory.

## 5 Conclusions

Our study highlights the correlation between SIRI levels and the risk of developing stress ulcers post‐stroke. Higher SIRI levels correlate with a greater risk of stress ulcers following stroke.

## Author Contributions

**Guojuan Chen**: conceptualization, investigation, formal analysis, data curation, methodology. **Liping Yan**: writing – review and editing, validation, visualization. **Peng Ding**: data curation, methodology, investigation. **Fanshu Zhao**: validation. **Ling Ling**: writing – review and editing, supervision. **Wei Yue**: conceptualization, funding acquisition, project administration, resources, supervision. **Yang Yang**: writing – original draft, validation, visualization. All authors read and approved the final manuscript.

## Funding

This study was supported by the Medical Science Research Project of Hebei Province (No. 20231793, to G.C.), the National Health Commission Research Center for Medical and Health Science and Technology Development (WKZX2023CZ0301, to W.Y.), and the Tianjin Key Medical Discipline Construction Project (Grant No. TJYXZDXK‐3‐014B, to W.Y.).

## Ethics Statement

This study was conducted in accordance with the Declaration of Helsinki. The study protocol was approved by the Institutional Review Board of Tianjin Huanhu Hospital (Approval No. 2022–158) on December 16, 2022. Informed consent was obtained from all participants.

## Data Availability

The data that support the findings of this study are available from the corresponding author upon reasonable request.

## References

[brb371867-bib-0001] Agarwal, R. , P. J. Tully , and R. Mahajan . 2024. “Cognitive Function in Atrial Fibrillation: A Narrative Review of Evidence and Mechanisms.” Heart and Mind 8, no. 2: 100–110. 10.4103/hm.HM-D-23-00075.

[brb371867-bib-0002] Alhazzani, W. , F. Alshamsi , and E. Belley‐Cote , et al. 2018. “Efficacy and Safety of Stress Ulcer Prophylaxis in Critically Ill Patients: A Network Meta‐Analysis of Randomized Trials.” Intensive Care Medicine 44, no. 1: 1–11. 10.1007/s00134-017-5005-8. PMC5770505 29199388

[brb371867-bib-0003] Ali, D. , M. E. Barra , and J. Blunck , et al. 2021. “Stress‐Related Gastrointestinal Bleeding in Patients With Aneurysmal Subarachnoid Hemorrhage: A Multicenter Retrospective Observational Study.” Neurocritical Care 35, no. 1: 39–45. 10.1007/s12028-020-01137-5. 33150575

[brb371867-bib-0004] Bresalier, R. S. 1991. “The Clinical Significance and Pathophysiology of Stress‐Related Gastric Mucosal Hemorrhage.” Journal of Clinical Gastroenterology 13, no. 2: S35–S43. 1885901

[brb371867-bib-0005] Chan, K. H. , K. S. Mann , E. C. Lai , H. Ngan , C. H. Tuen , and C. P. Yue . 1989. “Factors Influencing the Development of Gastrointestinal Complications After Neurosurgery: Results of Multivariate Analysis.” Neurosurgery 25, no. 3: 378–382. 10.1097/00006123-198909000-00010. 2771008

[brb371867-bib-0006] Chen, C. , L. Gu , and L. Chen , et al. 2020. “Neutrophil‐to‐Lymphocyte Ratio and Platelet‐to‐Lymphocyte Ratio as Potential Predictors of Prognosis in Acute Ischemic Stroke.” Frontiers in Neurology 11: 525621. 10.3389/fneur.2020.525621. PMC7868420 33569032

[brb371867-bib-0007] Chen, Y.‐F. , S. Qi , and Z.‐J. Yu , et al. 2023. “Systemic Inflammation Response Index Predicts Clinical Outcomes in Patients With Acute Ischemic Stroke (AIS) After the Treatment of Intravenous Thrombolysis.” The Neurologist 28, no. 6: 355–361. 10.1097/NRL.0000000000000492. PMC10627531 37027178

[brb371867-bib-0008] Chu, M. , Y. Luo , and D. Wang , et al. 2023. “Systemic Inflammation Response Index Predicts 3‐Month Outcome in Patients With Mild Acute Ischemic Stroke Receiving Intravenous Thrombolysis.” Frontiers in Neurology 14: 1095668. 10.3389/fneur.2023.1095668. PMC9946296 36846118

[brb371867-bib-0009] Deng, D. , H. Lei , and Z. Cao , et al. 2024. “Microbiome‐Gut‐Brain Axis Contributes to Patients and Bama Miniature Pigs With Acute Large Ischemic Stroke.” Frontiers in Neuroscience 18: 1378844. 10.3389/fnins.2024.1378844. PMC11272540 39071180

[brb371867-bib-0010] Ding, P. , G. Chen , and Y. Yang , et al. 2024. “Ischemic Insular Damage and Stress Ulcer in Patients of Acute Ischemic Stroke.” Brain and Behavior 14, no. 5: e3529. 10.1002/brb3.3529. PMC11095302 38747741

[brb371867-bib-0011] Ferro, D. , M. Matias , and J. Neto , et al. 2021. “Neutrophil‐to‐Lymphocyte Ratio Predicts Cerebral Edema and Clinical Worsening Early After Reperfusion Therapy in Stroke.” Stroke; A Journal of Cerebral Circulation 52, no. 3: 859–867. 10.1161/STROKEAHA.120.032130. 33517702

[brb371867-bib-0012] Gong, P. , Y. Liu , and Y. Gong , et al. 2021. “The Association of Neutrophil to Lymphocyte Ratio, Platelet to Lymphocyte Ratio, and Lymphocyte to Monocyte Ratio With Post‐Thrombolysis Early Neurological Outcomes in Patients With Acute Ischemic Stroke.” Journal of Neuroinflammation 18, no. 1: 51. 10.1186/s12974-021-02090-6. PMC7896410 33610168

[brb371867-bib-0013] Gong, Z. , J. Guo , and B. Liu , et al. 2024. “Mechanisms of Immune Response and Cell Death in Ischemic Stroke and Their Regulation by Natural Compounds.” Frontiers in Immunology 14: 1287857. 10.3389/fimmu.2023.1287857. PMC10808662 38274789

[brb371867-bib-0014] Gudeman, S. K. , C. B. Wheeler , J. D. Miller , L. G. Halloran , and D. P. Becker . 1983. “Gastric Secretory and Mucosal Injury Response to Severe Head Trauma.” Neurosurgery 12, no. 2: 175–179. 10.1227/00006123-198302000-00007. 6835500

[brb371867-bib-0015] Guo, H. , W.‐C. Liu , Y.‐Y. Sun , X.‐C. Jin , and P.‐P. Geng . 2026. “Neuroglia and Immune Cells Play Different Roles in Neuroinflammation and Neuroimmune Response in Post‐Stroke Neural Injury and Repair.” Acta Pharmacologica Sinica 47, no. 2: 273–289. 10.1038/s41401-025-01640-5. PMC12811257 40797113

[brb371867-bib-0016] Han, J. , L. Yang , Z. Lou , and Y. Zhu . 2023. “Association Between Systemic Immune‐Inflammation Index and Systemic Inflammation Response Index and Outcomes of Acute Ischemic Stroke: A Systematic Review and Meta‐Analysis.” Annals of Indian Academy of Neurology 26, no. 6: 655–662. 10.4103/aian.aian_85_23. PMC10666886 38022472

[brb371867-bib-0017] Han, X. , Y. Liu , G. Li , G. Tse , and T. Liu . 2023. “A Narrative Review on Prediabetes or Diabetes and Atrial Fibrillation: From Molecular Mechanisms to Clinical Practice.” Heart and Mind 7, no. 4: 207–216. 10.4103/hm.HM-D-23-00028.

[brb371867-bib-0018] Huang, L. 2022. “Increased Systemic Immune‐Inflammation Index Predicts Disease Severity and Functional Outcome in Acute Ischemic Stroke Patients.” The Neurologist 28, no. 1: 32–38. 10.1097/NRL.0000000000000464. PMC9812414 36125980

[brb371867-bib-0019] Ji, Y. , X. Xu , and K. Wu , et al. 2022. “Prognosis of Ischemic Stroke Patients Undergoing Endovascular Thrombectomy Is Influenced by Systemic Inflammatory Index Through Malignant Brain Edema.” Clinical Interventions in Aging 17: 1001–1012. 10.2147/CIA.S365553. PMC9259057 35814350

[brb371867-bib-0020] Kemp, W. J. , A. Bashir , H. Dababneh , et al. 2015. “Cushing's Ulcer: Further Reflections.” Asian Journal of Neurosurgery 10, no. 2: 87–94. 10.4103/1793-5482.154976. PMC4421974 25972936

[brb371867-bib-0021] Kim, J. Y. , J. Park , J. Y. Chang , S. H. Kim , and J. E. Lee . 2016. “Inflammation After Ischemic Stroke: The Role of Leukocytes and Glial Cells.” Experimental Neurobiology 25, no. 5: 241–251. 10.5607/en.2016.25.5.241. PMC5081470 27790058

[brb371867-bib-0022] Lee, S.‐H. , M. U.k Jang , and Y. Kim , et al. 2021. “The Neutrophil‐to‐Lymphocyte and Platelet‐to‐Lymphocyte Ratios Predict Reperfusion and Prognosis After Endovascular Treatment of Acute Ischemic Stroke.” Journal of Personalized Medicine 11, no. 8: 696. 10.3390/jpm11080696. PMC8399654 34442341

[brb371867-bib-0023] Li, Z.‐M. , L.e‐X. Wang , L.i‐C. Jiang , J.‐X. Zhu , F.‐Y. Geng , and F.u Qiang . 2010. “Relationship Between Plasma Cortisol Levels and Stress Ulcer Following Acute and Severe Head Injury.” Medical Principles and Practice 19, no. 1: 17–21. 10.1159/000252829. 19996614

[brb371867-bib-0024] Maclaren, R. , J. C. Dionne , and A. Granholm , et al. 2024. “Society of Critical Care Medicine and American Society of Health‐System Pharmacists Guideline for the Prevention of Stress‐Related Gastrointestinal Bleeding in Critically Ill Adults.” Critical Care Medicine 52, no. 8: e421–e430. 10.1097/CCM.0000000000006330. 39007578

[brb371867-bib-0025] Metz, C. A. , D. H. Livingston , J. S. Smith , G. M. Larson , and T. H. Wilson . 1993. “Impact of Multiple Risk Factors and Ranitidine Prophylaxis on the Development of Stress‐Related Upper Gastrointestinal Bleeding: A Prospective, Multicenter, Double‐Blind, Randomized Trial. The Ranitidine Head Injury Study Group.” Critical Care Medicine 21, no. 12: 1844–1849. 10.1097/00003246-199312000-00010. 8252888

[brb371867-bib-0026] Moran, K. T. , T. J. O'reilly , W. R. Furman , and A. M. Munster . 1987. “A Combined Regimen for the Prophylaxis of Curling's Ulcer.” American Surgeon 53, no. 10: 575–576. 2890321

[brb371867-bib-0027] Petrovic‐Djergovic, D. , S. N. Goonewardena , and D. J. Pinsky . 2016. “Inflammatory Disequilibrium in Stroke.” Circulation Research 119, no. 1: 142–158. 10.1161/CIRCRESAHA.116.308022. PMC5138050 27340273

[brb371867-bib-0028] Pikija, S. , L. K. Sztriha , and M. Killer‐Oberpfalzer , et al. 2018. “Neutrophil to Lymphocyte Ratio Predicts Intracranial Hemorrhage After Endovascular Thrombectomy in Acute Ischemic Stroke.” Journal of Neuroinflammation 15, no. 1: 319. 10.1186/s12974-018-1359-2. PMC6237008 30442159

[brb371867-bib-0029] Świtońska, M. , A. Słomka , P. Korbal , et al. 2019. “Association of Neutrophil‐to‐Lymphocyte Ratio and Lymphocyte‐to‐Monocyte Ratio With Treatment Modalities of Acute Ischaemic Stroke: A Pilot Study.” Medicina (Kaunas) 55, no. 8: 342. 10.3390/medicina55080342. PMC6680974 31284487

[brb371867-bib-0030] Venkatesan, S. , and B. Lucke‐Wold . 2025. “Mind the Gut: Navigating the Complex Landscape of Gastroprotection in Neurosurgical Patients.” World Journal of Gastroenterology 31, no. 8: 102959. 10.3748/wjg.v31.i8.102959. PMC11886513 40062336

[brb371867-bib-0031] Wang, H. , J. Li , and G. Wu , et al. 2024. “Activated Sympathetic Nerve Post Stroke Downregulates Toll‐Like Receptor 5 and Disrupts the Gut Mucosal Barrier.” Cell Reports Medicine 5, no. 10:101754. 10.1016/j.xcrm.2024.101754. PMC11513850 39383869

[brb371867-bib-0032] Wang, H. , Z. Yiqiang , J. Cai , and J. Guo . 2025. “Immune‐Mediated Blood‐Brain Barrier Disruption After Ischemic Stroke: Mechanisms and Therapeutic Targets.” Frontiers in Immunology 16: 1716041. 10.3389/fimmu.2025.1716041. PMC12727575 41451205

[brb371867-bib-0033] Wijdicks, E. F. , J. R. Fulgham , and K. P. Batts . 1994. “Gastrointestinal Bleeding in Stroke.” Stroke; A Journal of Cerebral Circulation 25, no. 11: 2146–2148. 10.1161/01.STR.25.11.2146. 7974536

[brb371867-bib-0034] Xiang, W. , X. Chen , W. Ye , J. Li , X. Zhang , and D. Xie . 2020. “Prognostic Nutritional Index for Predicting 3‐Month Outcomes in Ischemic Stroke Patients Undergoing Thrombolysis.” Frontiers in Neurology 11: 599. 10.3389/fneur.2020.00599. PMC7333017 32670192

[brb371867-bib-0035] Xie, X. , L. Wang , S. Dong , S. Ge , and T. Zhu . 2024. “Immune Regulation of the Gut‐Brain Axis and Lung‐Brain Axis Involved in Ischemic Stroke.” Neural Regeneration Research 19, no. 3: 519–528. 10.4103/1673-5374.380869. PMC10581566 37721279

[brb371867-bib-0036] Xu, Y. , J. Duan , D. Zhong , et al. 2025. “Identification of Autophagy‐Related Biomarkers in the Circulatory System of Ischemic Stroke.” Neurological Research 48, no. 8: 1090–1107. 10.1080/01616412.2025.2556537. 40898645

[brb371867-bib-0037] Yi, H. J. , J. H. Sung , and D. H. Lee . 2021. “Systemic Inflammation Response Index and Systemic Immune‐Inflammation Index Are Associated With Clinical Outcomes in Patients Treated With Mechanical Thrombectomy for Large Artery Occlusion.” World Neurosurgery 153: e282–e289. 10.1016/j.wneu.2021.06.113. 34217857

[brb371867-bib-0038] Zhou, Y. , Y. Zhang , M. Cui , Y. Zhang , and X. Shang . 2022. “Prognostic Value of the Systemic Inflammation Response Index in Patients With Acute Ischemic Stroke.” Brain and Behavior 12, no. 11: e2619. 10.1002/brb3.2619. PMC9226852 35588444

